# Data on public preferences for soil-based ecosystem services in Germany

**DOI:** 10.1016/j.dib.2022.108371

**Published:** 2022-06-10

**Authors:** Bartosz Bartkowski, Julian R. Massenberg, Nele Lienhoop

**Affiliations:** a Helmholtz Centre for Environmental Research – UFZ, Department of Economics, Permoserstraße 15, 04318 Leipzig, Germany; b Bochum University of Applied Science, School of Management & Economics, Am Hochschulcampus 1, 44801 Bochum, Germany

**Keywords:** Discrete choice experiment, Ecosystem services, Motivations, Nonmarket valuation, Stated preferences, Soil functions, Willingness to pay

## Abstract

This article describes the data from a discrete choice experiment survey into public preferences for soil-based ecosystem services. The survey was conducted online in June and July 2021 on a representative sample of 1500 German citizens. Four soil-based ecosystem services were included as attributes in the discrete choice experiment: climate regulation, drought protection, flood protection and clean drinking water. The collected data includes the stated choices from the choice experiment, measurements of knowledge about and awareness of soils’ contributions to human well-being, experience with droughts and floods, attitudes towards agriculture and environment and motivations for the stated choices as well as socio-demographic information. The dataset includes postcodes for all respondent, thus allowing for spatial analysis. The data can be used to investigate public preferences for soil-based ecosystem services and the underlying motivations.

## Specifications Table

**Table:** 

Subject	Agricultural Economics, Nature and Landscape Conservation
Specific subject area	Public preferences, motivations, self-assessed knowledge on soil-based ecosystem services
Type of data	CSV file
How the data were acquired	Online discrete choice experiment survey
Data format	Raw, long format
Description of data collection	The questionnaire was distributed via an existing, non-probability based online panel to a representative sample (n=1500) of the German population.
Data source location	Germany
Data accessibility	Repository name: BonaRes repository Data identification number: 10.20387/bonares-77fb-p034 Direct URL to data: https://doi.org/10.20387/bonares-77fb-p034 Questionnaire available at: https://metadata.bonares.de/smartEditor/rest/upload/cc787645-47d2-41a8-af10-b699a64d7b3b/SupplementalMaterial_cc787645-47d2-41a8-af10-b699a64d7b3b.zip

## Value of the Data

• Soil-related preference data are scarce despite soils’ importance for human well-being. We here provide comprehensive data on preferences, attitudes, motivations and self-assessed knowledge related to agricultural soils and the ecosystem services they provide.
• Researchers and actors in the agri-food system (e.g. policy makers, farmers’ associations, non-governmental organizations) can use the data to better understand public preferences towards agroecosystems.
• The data can be used to link preferences and more general attitudes, also in a spatially explicit manner.

## 1 Data Description

The data was collected in an online survey from a representative sample of the German population (n=1500). The survey consisted of a discrete choice experiment, questions on socio-demographic characteristics and a large set of questions related to general attitudes towards soils and agriculture. The choice experiment was used to investigate preferences and willingness to pay for soil-based ecosystem services. All data are in a format that does not allow to trace them back to individual respondents. The dataset in long format (CSV file), the original questionnaire in German (Soil_CE_questionnaire_final_de.pdf), an English translation (Soil_CE_questionnaire_final_en.pdf),the codebook (Soil_CE_survey_codebook.pdf) and information about the experimental design (Soil_CE_exp_design.pdf) are all available from the BonaRes repository: https://metadata.bonares.de/smartEditor/rest/upload/cc787645-47d2-41a8-af10-b699a64d7b3b/SupplementalMaterial_cc787645-47d2-41a8-af10-b699a64d7b3b.zip.

## 2 Experimental Design, Materials and Methods

The questionnaire was piloted on a sample of 50 respondents. The main survey was conducted in June/July 2021 on an existing non-probability based online panel curated by Innofact AG (https://innofact-marktforschung.de/).

The survey consisted of five parts:

1. Socio-demographic screening
2. Information and attitudes towards agricultural soils
3. Discrete choice experiment
4. Relevant experiences, general attitudes and motivations
5. Socio-demographic questions

In the following, parts 1 and 5 (socio-demographic questions) are presented together. Furthermore, the dataset contains information about total response times and response times for each choice set of the discrete choice experiment.

### 2.1 Socio-demographic questions

To ensure statistical representativeness, we used quotas related to:

• gender,
• age,
• level of education and
• residence in urban/rural area (determined via postcode).

Further socio-demographic data elicited in the survey include:

• residential situation (rented/owned flat/house),
• membership in and donations to environmental associations,
• relationship to agriculture (own or close others’ activity in farming or livestock husbandry),
• employment situation,
• household income,
• marital status,
• household size and presence of children below 18 in the household.

Table 1 presents an overview of the socio-demographic characteristics of the sample.

**Table 1:** Selected socio-demographic characteristics of the study sample and comparison with representativeness quotas

Variable	Sample value	Quota
Gender		
*Male*	755 (50%)	50%
*Female*	740 (49%)	50%
*Diverse*	5 (0%)	NA
Age (mean)	44.6	44.5
Level of education		
*Below abitur*	974 (65%)	60–70%
*Abitur or equivalent*	314 (21%)	15–20%
*Higher education*	212 (14%)	15–20%
Residence		
*Urban*	1222 (81%)	75–80%
*Rural*	278 (19%)	20–25%
Membership in environmental associations	153 (10%)	NA
Donations to environmental associations (last 12 months)	355 (24%)	NA
Relationship to agriculture		
*Farming*	173 (12%)	NA
*Livestock husbandry*	110 (7%)	NA
*None*	1261 (84%)	NA
Household monthly income		
*Below 1000€*	168 (11%)	NA
*1000–1500€*	197 (13%)	NA
*1500–2000€*	213 (14%)	NA
*2000–2500€*	230 (15%)	NA
*2500–3500€*	306 (20%)	NA
*3500–5000€*	266 (18%)	NA
*Above 5000€*	120 (8%)	NA

### 2.2 Information and attitudes towards agricultural soils

This part of the survey was developed with the help of soil scientists involved in the project. Following a general information about the importance of agricultural soils and the trade-offs involved in their management, respondents were asked a number of scale-based questions regarding:

• attitudes in the context of a set of recent public debates related to agriculture in Germany (organic farming, glyphosate ban, pesticide restrictions, small-scale family farms, modern technologies, fertilizer regulation),
• self-assessed awareness of the importance of soils for human well-being,
• self-assessed knowledge about the state of soils in their regions,
• general attitudes towards seven ecosystem services.

These more general questions were followed by more specific information about four soil-based ecosystem services (climate regulation, flood protection, drought protection, clean drinking water), including the maximum potential of an “average German soil” to provide each of these. Subsequently, the respondents were asked two scale-based questions about their perception of the importance of the four ecosystem services plus food production (i) from the perspective of society and (ii) from their own perspective.

### 2.3 Discrete choice experiment

Attributes were selected based on literature and expert opinion. Due to pandemic-related restrictions at the time of survey development, it was not possible to conduct focus groups to inform the attribute selection. Based on relevant literature [1,2] and iterative consultations with soil scientists from the BonaRes project, the following ecosystem services were identified as suitable attributes for the discrete choice experiment: climate regulation, flood protection, drought protection, and provision of clean drinking water.

Following similar studies conducted in Germany [3], [4], [5], an increase in annual household expenditures due to taxes needed to finance additional agri-environmental payment schemes as well as due to increases in food prices was used as payment vehicle.

All attribute levels were expressed in relative terms, i.e. how much of a given ecosystem service is provided compared to the maximum site-specific potential provision possible (given optimal management). As currently no spatially explicit data on the status quo provision of soil functions/soil-based ecosystem services is available, we defined the attributes for a “representative” German agricultural soil. We provided information about the maximum potential for such a representative German agricultural soil in the questionnaire.

The status quo alternative was defined based on expert opinion of soil scientists from the BonaRes project: for a representative German agricultural soil, it was set at 50% for climate regulation, 70% for each flood protection and drought protection, and 30% for clean drinking water. Based on these values, a set of evenly distributed levels for the other alternatives were defined (Table 2). The attribute levels for the price attribute were defined based on similar studies conducted in Germany [3,^6^,7].

**Table 2:** Attribute levels and corresponding variable names.

Attribute	SQ level	Levels
Climate regulation *(climate)*	50%	75%, 100%
Flood protection *(flood)*	70%	80%, 90%, 100%
Drought protection *(drought)*	70%	80%, 90%, 100%
Clean drinking water *(water)*	30%	50%, 75%, 100%
Increase in household expenditure per year *(price)*	0€	25€, 50€, 75€, 100€, 125€, 150€

The experimental design for the discrete choice experiment was generated with the help of the Ngene software, version 1.2.1 [8]. Following the Random Utility Theory (McFadden, 1974), we assumed that the utility function for alternative *i* in choice task *s* for respondent *n* is given by:

$$U_{ni}=V_{ni}+\;e_{ni}=\;\beta_{ni}x_{ni}+\;e_{ni}$$

where $V_{ni}$ is the observable utility component, $e_{ni}$ is the unobservable random utility component and $\beta_{ni}$ is the utility weight associated with attribute $x_{ni}$. Individuals are assumed to choose the alternative which provides the highest utility.

We generated a Bayesian D-efficient design [9] with eight two-alternative choice sets per respondent. The status quo option was not included in the design; it was added to each choice set afterwards. The D-efficient design requires prior parameter estimates (priors) which can be based on existing literature, derived from assumptions or obtained from piloting. Due to lack of existing studies, we first conducted a pilot study (pretest) with Bayesian priors close to zero and normal distribution. All ecosystem services were assumed to positively influence utility whereas price was assumed to have a negative effect on utility. In a next step, we used coefficient estimates (multinomial logit model) from this pretest as priors and the modified Fedorov algorithm [10] to generate the design for the main survey (see Table 3 for details on the priors used for the pretest and main survey).

**Table 3:** Attribute priors used for the Bayesian D-efficient design for the pilot/pretest and main survey.

Attribute	Pretest: initial priors (mean, standard deviation)	Main survey: revised priors (mean, standard deviation)
Climate regulation *(climate)*	0.0001, 0.00003	0.0099, 0.0572
Flood protection *(flood)*	0.0001, 0.00003	0.0121, 0.0566
Drought protection *(drought)*	0.0001, 0.00003	0.0193, 0.0369
Clean drinking water *(water)*	0.0001, 0.00003	0.0208, 0.0235
Increase in household expenditure per year *(price)*	-0.01, 0.003	-0.013, 0.0120

**Fig. 1:**
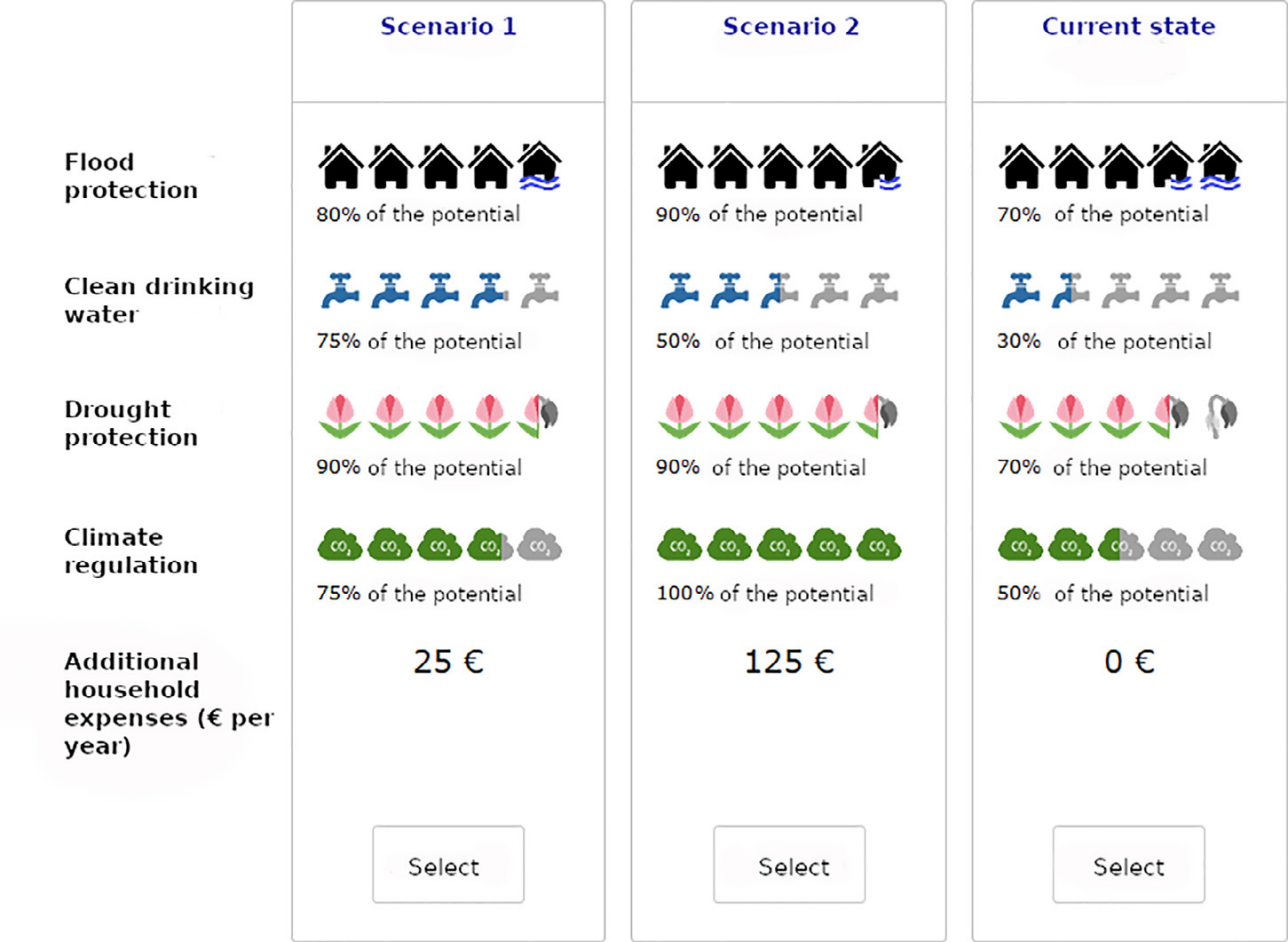
Example choice set.

We generated 30 blocks, which were then randomly assigned to respondents. To each block, a constant choice set was added to allow for validation of simulation results, which resulted in nine choice sets per individual in the final design (the constant choice set was selected from the pretest design). The order of choice tasks was fixed for each block; the validation task was always included as the fifth task. The order of the ES attributes was randomized across choice tasks. All code and data related to the experimental design are available from GitHub (https://github.com/BartoszBartk/soil-ce). An example choice set can be found in Fig. 1.

### 2.4 Relevant experiences, general attitudes and motivations

To capture factors that may affect and help to better understand the preferences elicited in the discrete choice experiment part, the questionnaire included the following questions:

• experience (own or close others’) with floods and droughts,
• perception of responsibility of different actors for sustainable agricultural soil management,
• a modified version of Massenberg's [11] range of items related to motivation behind the choices, with reference to aspects of identified needs and values hierarchies, social or individual orientation of preferences (so-called We-preferences and I-preferences [12]), Ajzen's [13] concept of perceived behaviour control (see also Spash et al. [14]) and the German translation of the New Ecological Paradigm Scale [15,16],
• Inclusion of Nature in Self (INS) [17] and Inclusion of Community in Self scales (ICS) [18,19].

## Ethics Statements

All participants involved in the study provided their written informed consent (to participate in this study) according to the European General Data Protection Regulation (GDPR). Participation was voluntary and could be withdrawn at any time. Anonymity of the data is guaranteed as no personal identifiable information about the respondents was collected.

## CRediT Author Statement

**Bartosz Bartkowski:** Conceptualization, Methodology, Data Curation, Writing – original draft and review & editing, Funding acquisition; **Julian Massenberg:** Conceptualization, Methodology, Writing – review & editing; **Nele Lienhoop:** Conceptualization, Methodology, Writing – review & editing.

## Declaration of Competing Interest

The authors declare that they have no known competing financial interests or personal relationships that could have appeared to influence the work reported in this paper.

## Data Availability

German public's preferences for soil-based ecosystem services (discrete choice experiment) (Original data) (BonaRes Repository).
